# Role of paraoxonase 2 (PON2) as a potential biomarker and therapeutic target in cancer treatment

**DOI:** 10.1007/s00432-025-06282-y

**Published:** 2025-08-12

**Authors:** Vinayak Agarwal, Matthew Cheesman, Alison Haywood, Sohil Khan, Shailendra Anoopkumar Dukie

**Affiliations:** 1 School of Pharmacy and Medical Sciences, Griffith University, Gold Coast, Australia; 2 Mater Research Institute, The University of Queensland, Brisbane, Australia

**Keywords:** Paraoxonase 2 (PON2), Oxidative stress, Cancer biomarker, Therapeutic target, Chemotherapy resistance, Prognostic marker

## Abstract

**Purpose:**

Paraoxonase 2 (PON2), a mitochondrial antioxidant enzyme, is widely expressed across body tissues and plays vital roles in cellular defence. Growing evidence links its dysregulation to oxidative stress and cancer progression. This narrative review aims to elucidate PON2’s enzymatic characteristics and explore its potential modulation as an innovative therapeutic avenue for combating cancer.

**Methods:**

This narrative review provides a comprehensive evaluation of the roles of paraoxonase 2 in cancer, with a focus on its regulatory mechanisms and potential utility as a biomarker for targeted therapeutic interventions. A comprehensive analysis of the scientific literature from 1924 to 2024 was carried out, with studies retrieved from PubMed, Web of Science, and Scopus. Following a comprehensive screening process, 27 studies were included in the review.

**Results:**

The outcomes underscore PON2's complex involvement in cancer biology, spanning tumour initiation, progression, and therapeutic resistance. The antioxidant attributes of PON2 play a critical role in helping cancer cells combat chemotherapy by allowing them to sustain oxidative stress. PON2 regulates essential features of the cancer cell lifecycle, such as invasion, migration, and proliferation. Its impact on apoptosis makes it promising as a biomarker for prognosis and diagnostics as well as a therapeutic target, especially for patients with drug-resistant cancers.

**Conclusion:**

This review highlights PON2’s involvement in carcinogenesis in detail. Future research should focus on elucidating the molecular mechanisms regulating PON2 and exploring its potential as a target for the development of effective cancer therapies.

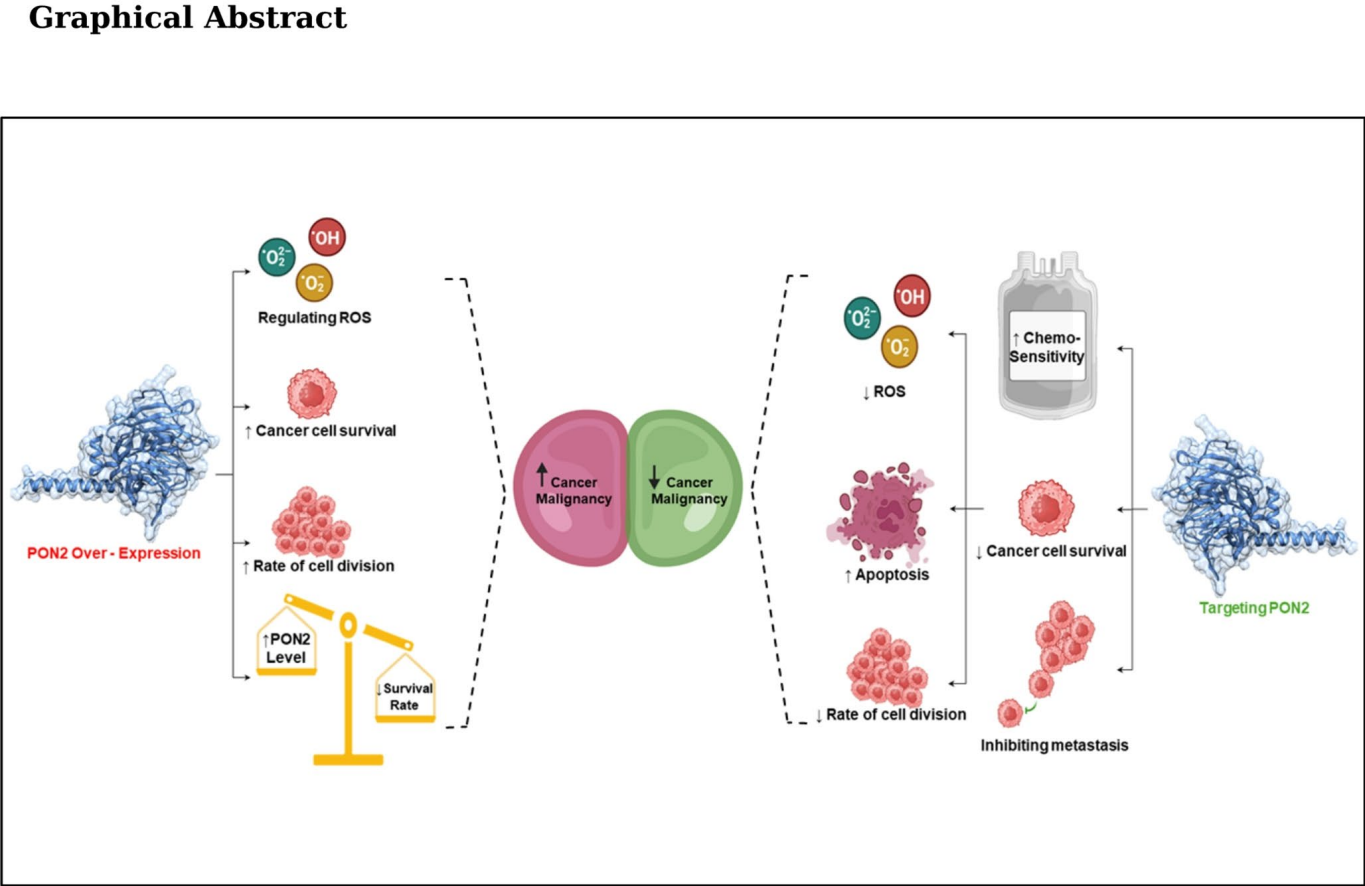

## Background

Paraoxonase-1 (PON1), PON2 and paraoxonase-3 (PON3) are esterase enzymes that are highly conserved across and between species (Teiber et al. 2018; Lewoń-Mrozek et al. 2024). PON2 is the oldest member of this family, as determined by phylogenetic studies, with PON1 and PON3 descending from PON2 (Chen et al. 2004). The three paraoxonase genes have been localised on the long arm of chromosome 7 in humans and chromosome 6 in mice (She et al. 2012; Campagna et al. 2024; Ayan et al. 2024). While PON2 is widely distributed and not present in blood plasma, PON1 and PON3 are predominantly expressed in the liver and are related to high-density lipoprotein (HDL) (Ng et al. 2001; Kulka 2016). PON2 has been detected in its mRNA and protein forms in several tissues, including the brain where PON1 and PON3 are not expressed (Giordano et al. 2011; Costa et al. 2014). The lungs and small intestine possess the highest levels of PON2, followed by the heart and liver (Marsillach et al. 2008). While the testis, kidney, and brain contain the lowest levels of this isoform. The physiological functions and native substrates of paraoxonase are yet to be identified despite their heterogeneous gene expression (Draganov et al. 2005; Richter et al. 2009).

Lactones are believed to be endogenous substrates of the PON enzymes based on their intrinsic lactonase activity (Grdic Rajkovic et al. 2011). Subcellular localization investigations have revealed that PON2 is present in the mitochondria, endoplasmic reticulum, and perinuclear area (Horke et al. 2007). PON2 performs a critical role in shielding the elements of the membrane from peroxidation at the plasma membrane by operating as a transmembrane protein whose enzymatic domain exposes the extracellular compartment (Hagmann et al. 2014). Mitochondria play an essential function in both cell life and death since they are necessary for preserving certain physiological, biochemical, and architectural aspects of cells (Rasheed et al. 2017). PON2's positioning in the mitochondria reveals that it plays a cytoprotective and antioxidative role, and its absence triggers mitochondrial malfunction (Devarajan et al. 2011). PON2 possesses considerably higher lactonase activity as compared to PON1 and PON3, while also exerting antioxidant activities. It is also possible that PON2 has anti-inflammatory properties, due to its lactonase activity (Teiber et al. 2008). Indeed, the inhibition of PON2 in intestinal epithelial cells impairs inflammatory processes and compromises mucosal integrity (Précourt et al. 2012). Interestingly, a lactonase-deficient PON2 mutant (PON2-Asn254Ala/Asn323Ala) retains its anti-inflammatory properties (Stoltz et al. 2009). Additionally, PON2 mutant mice display an enhanced macrophage inflammatory response in vivo (Ng et al. 2006). Since PON2 has no known targets or substrates within any specific physiological environments, the mechanism by which it delivers its anti-inflammatory action is still poorly understood.

An integrated, multistep mechanism that is influenced by cholesterol levels, oxidative stress, and genetic factors appear to govern the transcription of the PON2 gene (Rosenblat et al. 2004; Shiner et al. 2006). Lower transcriptional levels have been linked to recurrent abortions since PON2 has insufficient antioxidant activity (Dikbas et al. 2018). PON2 expression is known to be upregulated by several transcription factors including activator protein-1 (AP-1), sterol regulatory element-binding protein 2 (SREBP-2), and peroxisome proliferator-activated receptor c (PPARc). As an example, sex steroids govern hormones in an intricate manner which substantially impacts PON2 transcription. Research has revealed that female mice contain higher levels of PON2 in their peripheral tissues and central nervous systems compared to males. Such an outcome could potentially have ramifications for the aetiology of several neurodegenerative diseases along with other pathological conditions (Giordano et al. 2011; Siddiqui et al. 2016). The positive modulatory effects of oestrogens have been suggested to be the root cause of elevated levels in females (Leranth et al. 2000; Kitamura et al. 2009; Bwire 2020). Within both male and female striatal astrocytes, estradiol has been demonstrated to improve PON2 mRNA as well as protein levels in a time-sensitive and concentration-dependent manner. Such an impact is probably due to the stimulation of the oestrogen receptor alpha. Furthermore, ovariectomised female mice show lower levels of PON2 mRNA and protein in the liver and brain as compared to males (Cheng and Klaassen 2012; Giordano et al. 2013). Males appear to be more prone to oxidative stress in different organs, which could be explained by this variation in PON2 expression across genders.

PON2-specific lactonase and arylesterase activities appear to be impaired by nonsteroidal anti-inflammatory drugs (NSAIDs). They include drugs such as tenoxicam and diclofenac sodium, where in vitro studies in U937 cells, show significant decreases in lactonase activity of PON2 (Solmaz Avcıkurt and Korkut 2018). It has also been suggested that increased cholesterol levels suppress PON2 expression (Dikbas et al. 2017). Consequently, hypocholesterolemic medications may lead to increases in PON2 expression, and this has been demonstrated with atorvastatin which stimulates PON2 expression in various cell types (Shiner et al. 2007). Together, these findings indicate that PON2 regulation and its roles in inflammation and oxidative stress provide avenues of investigation into the genetic, hormonal, and pharmacological regulation of PON2. In turn, this may result in novel treatment options for associated disorders involving gender-specific expression discrepancies.

The PON family of enzymes, comprising PON1, PON2, and PON3, are highly expressed in various cancer cells and play critical roles in promoting cell survival and metastasis (Witte et al. 2012). Due to their significant involvement in cancer progression, PON enzymes have emerged as attractive therapeutic targets. Notably, these isotypes share a high degree of sequence similarity, ranging from 81 to 95% (Bacchetti et al. 2021), and are known to mitigate oxidative stress a hallmark of malignancies, while regulating cancer cell progression. Although initial research on PON2 primarily focused on its antioxidant protective roles in atherosclerosis, recent studies have increasingly implicated it in cancer pathogenesis. Elevated PON2 expression has been associated with poorer prognosis in patients with multiple solid tumours (Shi and Jin 2010). Advanced analytical techniques, including Western blotting, immunoprecipitation, and surface plasmon resonance, have further elucidated PON2’s antioxidant activity in cancer cells.

Functionally, PON2 inhibits the accumulation of superoxides and preserves mitochondrial respiratory chain integrity by stabilizing reactive semi-ubiquinone CoQ10 species, thus preventing excessive reactive oxygen species (ROS) generation (Devarajan et al. 2011). This interaction is particularly significant given the central role of mitochondria in carcinogenesis (Lass and Sohal 1999).

Importantly, PON2 appears to contribute to cancer stem cell (CSC) biology. CSCs require a hypoxic microenvironment to maintain their pluripotency and differentiation potential. PON2 is overexpressed in various CSC lines and is positively correlated with Oct-4, a key transcription factor governing stemness (Shi and Jin 2010). Hypoxia-inducible factor 1 (HIF-1) further induces PON2 expression, promoting mitochondrial antioxidant effects that support CSC maintenance (Tang et al. 2014). This suggests that targeting PON2, possibly through the induction of ROS overload, could sensitize resistant cancer cells and enhance the efficacy of existing chemotherapies.

In gastric cancer, elevated PON2 expression correlates with aggressive clinicopathological features such as diffuse tumor type, advanced stages, invasion, and metastasis. Survival analyses have demonstrated that higher PON2 levels are linked to significantly reduced patient survival, while silencing PON2 inhibits cancer cell migration, proliferation, and metastatic potential (Bacchetti et al. 2017).

Given its widespread tissue distribution and mitochondrial localization, PON2 serves as a crucial antioxidant and a biomarker for oxidative stress susceptibility. Therapeutic strategies aimed at reducing PON2 expression in tumor cells hold promise, as further understanding of the regulatory mechanisms governing PON2 upregulation may yield novel insights into carcinogenesis and facilitate the development of effective interventions. This narrative review aims to comprehensively synthesize existing literature on PON2 regulation in cancer, enhancing understanding of its multifaceted role and exploring its potential as a prognostic marker and therapeutic target.

## Methodology

This review adopts a narrative approach to synthesise key findings related to the role of PON2 as a potential biomarker and therapeutic target in cancer treatment. While not following the formal protocol of a systematic review, a structured and transparent method was employed to ensure a comprehensive and balanced discussion. The relevant literature was identified through targeted searches of electronic databases, including PubMed, Web of Science, and Scopus, and were accessed for the study. Using a combination of specific keywords, search terms were synthesised using Boolean operators (AND, OR, and NOT) (Table 1).

**Table 1:** Search terms used for retrieving data from the listed databases for the study

CODE	Search terms
A	(Paraoxonase 2 AND cancer OR tumor OR carcinoma OR cancer therapeutic) AND (PON2)
B	(Paraoxonase 2 AND oxidative stress OR ROS AND cancer OR tumor OR carcinoma) AND (PON2)
C	(Paraoxonase 2 AND cellular proliferation OR cell division AND cancer OR tumor OR carcinoma) AND (PON2)
D	(Paraoxonase 2 AND antiapoptotic effects OR pro survival effects AND cancer OR tumor OR carcinoma) AND (PON2)
E	(Paraoxonase 2 AND biomarker OR diagnostic marker OR prognostic marker OR biological marker AND cancer OR tumor OR carcinoma) AND (PON2)
F	(Paraoxonase 2 AND therapeutic target OR drug target OR cancer treatment AND cancer OR tumor OR carcinoma) AND (PON2)
G	(Paraoxonase 2 AND metastasis OR apoptosis OR TME OR tumor microenvironment AND cancer OR tumor OR carcinoma) AND (PON2)
H	(Paraoxonase 2 AND mitochondria OR mitochondrial respiration OR electron transport chain OR CoQ10 AND cancer OR tumor OR carcinoma) AND (PON2)
I	(Paraoxonase 2 AND mitochondria OR mitochondrial respiration OR electron transport chain OR CoQ10) AND (PON2)
J	(Paraoxonase 2 AND metastasis OR apoptosis OR TME OR tumor microenvironment) AND (PON2)
K	(Paraoxonase 2 AND antiapoptotic effects OR pro survival effects) AND (PON2)
L	(Paraoxonase 2 AND therapeutic target OR drug target OR cancer treatment) AND (PON2)
M	(Paraoxonase 2 AND biomarker OR diagnostic marker OR prognostic marker OR biological marker) AND (PON2)
N	(Paraoxonase 2 AND cellular proliferation OR cell division) AND (PON2)
O	(Paraoxonase 2 AND oxidative stress OR ROS) AND (PON2)

^†^The search terms in this table were used to retrieve relevant literature from selected databases for the study on paraoxonase 2 (PON2) in cancer. Boolean operators (AND, OR) were applied to refine the search strategy, ensuring comprehensive coverage of PON2’s role as a potential biomarker and therapeutic target in cancer research

The search was limited to articles in English between 1924 and 2024. The present dataset encompasses a wide array of studies, ensuring a robust and thorough representation of the field's scholarly output over this extensive timeline. Peer-reviewed original research articles integrating human cancer cell lines or animal models that have investigated PON2's role in cancer have been included in this narrative review, in addition to the involvement of PON2 as a biomarker or therapeutic target, enzyme expression and regulation, significance in the development of cancer, and the enzyme’s potential for treatment. Review articles, meta-analyses, editorials, case reports, conference abstracts, non-peer-reviewed literature, and studies extraneous to cancer models or those not specifically focused on PON2 have been excluded. Studies published in languages other than English and those without pertinent biomarkers or therapeutic findings were included. Data extraction and quality assessment for the screened studies using the covidence software was employed and adhered to uphold the quality of the research throughout the data screening.

## Results

A total of 2322 studies had been retrieved from three prominent databases: Scopus (*n* = 245), Web of Science (*n* = 1032), and PubMed (*n* = 1045). To facilitate a comprehensive search of relevant literature, the studies were discovered by employing specific search terms designed using Boolean operators. Covidence software had been employed to monitor the screening process, automatically removing 1920 duplicate studies, while six studies had been excluded manually. Following the removal of duplicates, 305 studies were ruled out as they were not relevant to the scope of this review, including those unrelated to PON2, cancer models, or therapeutic/biomarker contexts. Once the title and abstract screening had been performed. A total of 91 studies then remained for full-text review to assess eligibility. During the full-text review phase, 64 studies were excluded for being extraneous (*n* = 36), offering wrong indication (*n* = 17), or having the wrong study design (*n* = 11). As a result, 27 studies met the inclusion criteria and were ultimately included in the review. Data extraction for these studies has also been conducted using covidence software.

**Fig. 1:**
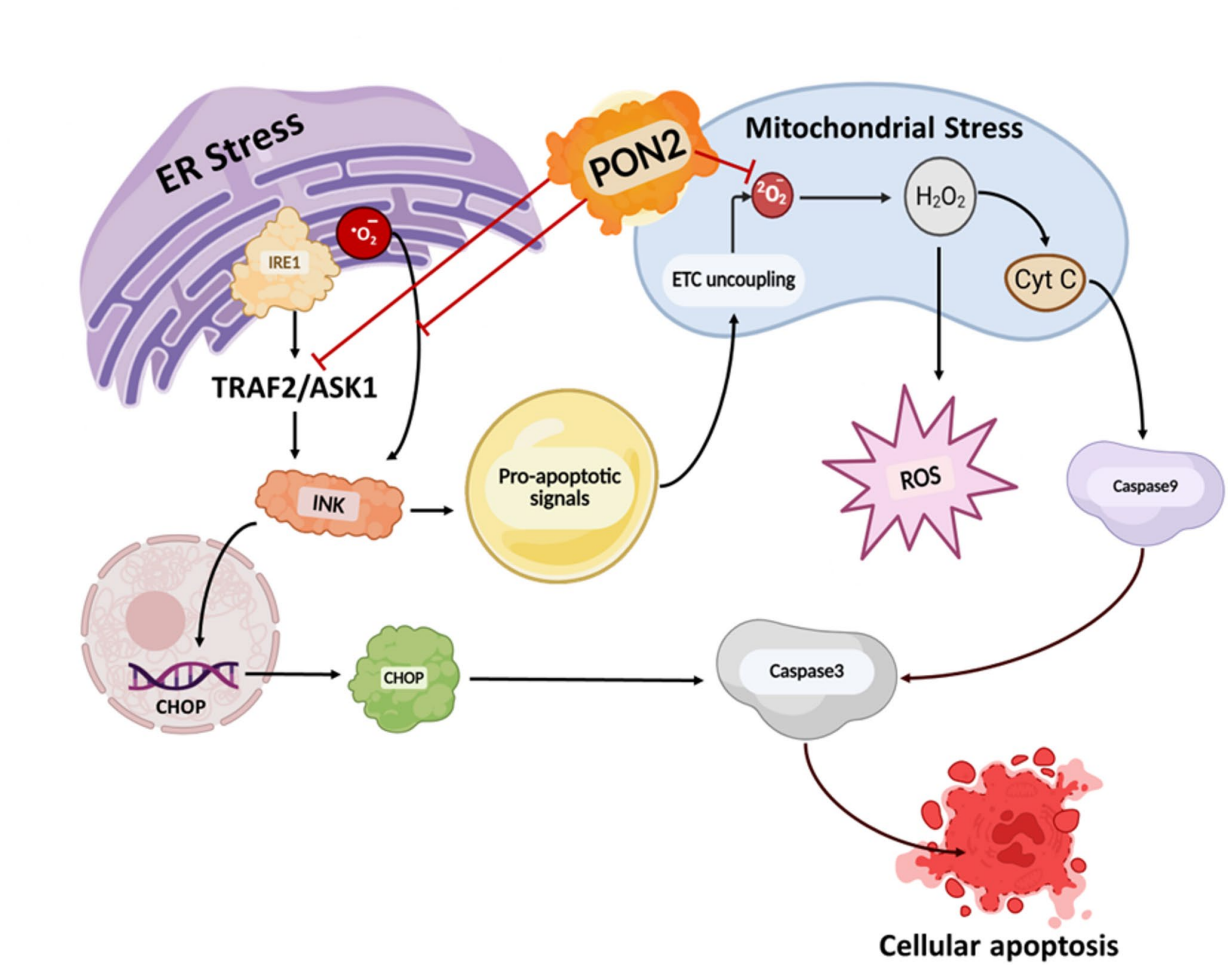
PON2 as a key regulator of oxidative stress and apoptosis in cancer progression. Abbreviation: PON2: paraoxonase 2; Cyt C: cytochrome c; ROS: reactive oxygen species; CHOP: C/EBP homologous protein; ER: endoplasmic reticulum; INK: INK4 family of cyclin-dependent kinase inhibitors; IRE1: Inositol-Requiring Enzyme 1; TRAF2: TNF Receptor–Associated Factor 2; ASK1: Apoptosis Signal-Regulating Kinase 1

Collectively, these studies have explored the regulatory role of PON2 in carcinogenesis, particularly highlighting its potent antioxidative capacity and its involvement in mechanisms that promote cancer cell survival. The markedly high antioxidative activity of PON2 prevents electron transport chain (ETC) uncoupling and mitigates mitochondrial oxidative stress during metastasis, a stage characterised by accelerated cellular proliferation and excessive accumulation of ROS. Furthermore, PON2 disrupts the cellular apoptotic machinery by inhibiting the INK4 family of cyclin-dependent kinase inhibitors (INK proteins). These INK proteins function as tumour suppressors by blocking the phosphorylation of the retinoblastoma protein (Rb), thereby arresting the cell cycle at the G1 phase and preventing uncontrolled proliferation. If INK proteins remain active, they can initiate apoptotic signalling by (i) acting as transcriptional regulators to induce the expression of C/EBP Homologous Protein (CHOP), an established marker of endoplasmic reticulum (ER) stress that drives caspase-3 activation under prolonged oxidative stress, and (ii) transmitting pro-apoptotic signals to the mitochondria, triggering cytochrome c release and subsequent activation of caspase-9 and caspase-3, culminating in apoptosis (Fig. 1) (Witte et al. 2012). Through its regulatory expression, PON2 effectively circumvents both oxidative stress and pro-apoptotic pathways, thereby facilitating cancer cell survival, as discussed in detail in the following sections.

### PON2 expression and its role in tumorigenic microenvironments

Recently, the significance of PON2 in cancer has garnered increasing attention, with emerging evidence elucidating its complex involvement in tumour biology and therapeutic responsiveness in cancer (Kamal et al. 2024). PON2 expressions and functions appear to be closely linked to cancer cell survival and metastasis across multiple cancer types. Ongoing research has uncovered several regulatory pathways controlling PON2 expression and supports its potential as a therapeutic target beyond its role as a diagnostic marker (Table 2).

**Table 2:** PON2 Expression modulation in response to therapeutic interventions in cancer systems

Study ID	Investigated cancer type	Cell line/animal model	Effects on PON2 expression
Bacchetti et al. (2022)	Advanced-stage bladder cancer and colorectal adenocarcinoma	T24 and Caco2	↑ PON2 in T24 cells after incubation with CSE at all tested conc. at *p* < 0.001
			↑ PON2, 2.5-time higher in T24 cells treated with the lower CSE concentration (10 µg GAE/mL) compared to untreated cells
Ng et al. (2001)	Cervical cancer	HeLa	PON2 mRNA is ubiquitously expressed in nearly all human tissues
			↑ PON2 expression in liver, lung, placenta, testis, and heart
			↑ PON2; Upon treating HeLa-Tet-PON2 cells for 48 h with 2 g/ml doxycycline
Zhao et al. (2016)	–	A549, HCT116, NHBE	↑ PON2 in tumour tissues (in 8 samples) compared to adjacent normal tissues
			↓ PON2 in NSCLC cell lines A549 and NCI-H1299 using shRNA PON2 knockdown
		In vivo model: C57BL/6 mice or in 6-week-old athymic nude mice	PON2 acts as a lactonase to cleave C12, a quorum-sensing molecule
Xie et al. (2021)	Thyroid cancer	BCPAP, BHP5-16, TPC-1 and CGTH-W3	TargetScan predicted PON2 as a potential target of miR-376a-3p in thyroid cancer
			miR-376a-3p ↓ PON2
			RT-qPCR, Western blot, and immunocytochemistry showed ↓ PON2 in BCPAP and TPC-1 cells transfected with miR-376a-3p mimic
Witte et al. (2011)	Leukaemia and lung carcinoma	EA hy 926 cells, HEK293, K562, A549	↑ PON2 reduces ER stress-induced cell death and mitochondrial superoxide production
			↑ PON2 may protect against atherosclerosis by preventing cell death in vascular cells and macrophages
			↑ PON2 levels (2–fourfold), are found in tumours from endometrium/uterus, liver, kidney, lymphoid tissues, and urinary bladder
			↑ PON2 reduces ER stress-induced apoptosis by lowering CHOP mRNA and protein levels
			JNK inhibition with SP600125 lowers tunicamycin-triggered CHOP induction in naive cells but not in PON2-overexpressing cells
			JNK knock-down by siRNA decreases CHOP induction in naive cells but not in PON2-overexpressing cells
Whitt et al. (2023)	LLC and lung adenocarcinoma cells	A549, NCIH1299, and HEK-293 T cells	↑ PON2 was stably reduced in LLC cells using RNAi
			LLC cells with ↓ PON2 displayed a higher percentage of cells in G1 phase and a lower proportion in S phase
		In vivo model: KrasLSL-G12D mouse model of primary lung tumorigenesis	↑ PON2 expression is increased in human lung adenocarcinoma cells
			↓ PON2 in A549 and NCI-H1299 cells arrested cell cycle progression at the G1 phase
			↓ PON2 in HEK-293 T and HBE cells conferred resistance to C12
Wang et al. (2019)	GC	MGC-803, AGS, BGC-823, MKN45 and SGC-7901	↑ PON2 mRNA and ↑ PON2 expression in GC tissues compared to adjacent normal tissues (*p* < 0.001)
			↓ PON2 expression in 9 of 37 adjacent normal gastric mucosal tissues (*p* = 0.003)
			Kaplan Meier Method and Log-Rank Test: ↑ PON2 expression had significantly shorter overall survival vs those with ↓ PON2 expression (*p* < 0.001)
			Multivariate Cox Proportional Hazards Regression Analysis: ↑ PON2 mRNA and protein in GC tissues and their association with clinical progression
			Loss-of-Function Study: In MKN45 and SGC-7901 cells, which had relatively ↑ PON2 expression
			si-PON2 Transfection: Led to ↓ PON2 expression in MKN45 and SGC-7901 cells
Tseng et al. (2017)	GBM	U87, GBM8401, and DBTRG05MG	PON2 was downregulated, and p27 was upregulated at 24 h by VPA in U87, GBM8401, and DBTRG-05MG cells
			VPA treatment (5 and 10 mM) for 24 to 72 h ↓ PON2 mRNA and protein levels are dose-dependent, confirmed by RT-PCR and Western blot
		In vivo model: BALB/c nude mice	VPA ↓ PON2 expression, reversed by 1-benzoyl-3-phenyl-2-thiourea in U87, GBM8401, and DBTRG-05MG cells
			↓ PON2 expression and ↑ Bim expression by VPA in tumours of GBM8401 cells
			↓ PON2 protein expression in PON2-silenced cells (U87 and GBM8401)
Sukketsiri et al. (2013)	Hepatoma	HepG2 cells	↓ PON2 activity in HepG2 cell lysates with lead acetate exposure in a concentration- and time-dependent manner
			Significant ↓ PON2 activity as early as 4 h at 0.5 mg/ml (1.32 mM) lead acetate
			Longer exposure (over 24 h) led to significant ↓ PON2 activity at 0.05 mg/ml (0.13 mM)
			RT-PCR revealed significant ↑ PON2 mRNA levels with lead acetate exposure
			↓ PON2 activity is reversed by calcium addition
Shakhparonov et al. (2018)	Liver carcinoma, glioblastoma, lung cancer, ovarian carcinoma, lung carcinoma, leukaemia, fibrosarcoma	U87-MG, MRC5-V2, SKOV3, A549, HepG2 and, HT1080	↑ PON2 levels were found in liver cancer and brain cancer (grade 1 and 3 gliomas and grade 4 glioblastoma)
			Lowest expression levels of PON2 were observed in leukaemia (myeloid leukaemia and B-cell lymphoma)
			Genomic DNA Analysis for PON2 Mutations:
			Amplification of the PON2 gene is typical in glioblastoma
			Deletion of the PON2 gene is usually observed in leukaemia
			PON2 helps liver and brain cancer cells adapt to toxic and nutrient-poor environments
			Tumours with ↑ PON2 expression may develop due to gene amplification
Schwarzer et al. (2015)		HEK293T cells	RNAseq data shows ↑ PON2 levels in WT and DKOR MEF, ↓ levels in DKONR MEF
			↑ PON2 expression in C12-sensitive WT and DKOR MEF, ↓ in nonresponsive DKONR MEF
		In vivo model: MEF—Wild type, Bax/Bak DKO MEF	DKONR MEF treated with adenovirus-GFP: ↓ PON2 levels
			DKONR MEF treated with adenovirus-hPON2: ↑ PON2 levels
			HEK293T cells showed ↓ expression of PON2,
Schiavoni et al. (2024)	Renal cell carcinoma	ccRCC-786 and Caki-1	↓ PON2 was measured at both mRNA and protein levels using real-time PCR and Western blot analysis
			pLKO.1–647 plasmid effectively ↓ PON2 expression at both mRNA and protein levels in 786-O and Caki-1 cell lines
			786-O Cells; ↓ PON2 mRNA and protein levels with pLKO.1–647 plasmid (0.41 Â ± 0.07) and (0.42 Â ± 0.072) respectively
			Caki-1 cells; ↓ PON2 mRNA and protein levels with pLKO.1–647 plasmid (0.48 Â ± 0.005) and (0.39 Â ± 0.003) respectively
Que et al. (2022)	AITL	54—FFPE tissue samples	Rhoa G17V-expressing CD4 + T cells exhibited significant ↑ PON2 expression
			RHOA G17V mutation activated PI3K/Akt/IKK signalling, ↑ NF-κB activity and PON2 expression
			NF-κB inhibition abolished PON2 expression in Rhoa G17V-expressing CD4 + T cells
			PON2 knockdown leads to ↓ serum levels of TC cell-produced pro-inflammatory cytokines (IL-6 and IL-21)
Parween et al. (2022)	Neuroblastoma	IMR-32	↓ PON2 protein expression, at 50 µM and 100 µM conc. of CPF
			PA exposure leads to ↑ PON2 protein expression at higher conc
			No paraoxonase activity was detected in IMR-32 cells treated with any concentrations of CPF and PA
Pan et al. (2021)	B-ALL	B-ALL cells	At the time of relapse, B-ALL patient samples ↑ PON2 mRNA levels compared to diagnosis (*p* = 0.041)
			↑ PON2 mRNA levels in Ph + B-ALL patient samples compared to normal pre-B cells
		In vivo model: PON2 ^− −^/^− −^, mice (26) were backcrossed to wild- type C57BL/6 J for more than 8 generations	PON2 expression levels do not correlate with clinical outcomes in mature B cell lymphomas and multiple myeloma, indicating a specific connection with B-ALL
			Loss of PON2 ↑ cell cycle checkpoint molecules Arf and p21, leading to G0/1-cell cycle arrest
Nagarajan et al. (2017)	PDAC	PANC1, AsPC-1, MiaPaCa-2, SU.86.86, HPNE-hTERT, and HPNE-hTERT E6/E7/st cell line	PON2 knockdown inhibited tumour formation in two mouse models of PDAC tumour
			Ectopic expression of p53 in the p53-deficient PDAC cell line AsPC-1; ↓ PON2 mRNA and protein levels
		In vivo model: Athymic nude mice (both male and female) (NCr nu/nu, 8 weeks old) 2 – mouse models: a subcutaneous tumour xenograft model and an orthotopic pancreatic tumour xenograft model	p53 knockdown; ↑ PON2 mRNA and protein levels, reducing p53 enrichment on PON2 promoter (HPNE-hTERT cells)
			p53 acts as a direct transcriptional repressor of PON2, and its inactivation is key to ↑ PON2 in PDAC
			PON2 regulates glucose metabolism by mediating the interaction between STOM and GLUT1, affecting glucose transport in PDAC cells
Krüger et al. (2016)	Oral squamous cell cancer and leukaemia	SCC-4 and PCI-13, K562, HUVEC and EA.hy 926 cells	Wnt3 ↑ PON2 expression in K562 cells
			HUVEC cells, both Wnt3 and Wnt5 ↑ PON2 protein levels
			SCC4 cells with ↑ PON2 levels had ↑ endogenous Lef-1 activity compared to PCI-13 cells with low PON2 expression
			β-catenin activation did not ↑ PON2 levels in SCC4 cells, while it significantly ↑ PON2 in PCI-13 cells
			K562 leukaemia cells were treated with VPA (1 mM) for 3 days; doubled PON2 mRNA expression after 2 days and ↑ PON2 protein levels after 3 days
			SB216763 induced PON2 promoter activity significantly after 2 days, correlating with ↑ PON2 mRNA and protein levels in K562 cells
Campagna et al. (2023)	Triple-negative breast cancer	MCF-7 (ER +), SKBR-3 (HER2 +) and MDA-MB-231 (TNBC)	Silencing PON2 expression in the cell line transfected with pLKO.1–647 plasmid, ↓ PON2 mRNA (*p* = 0.041), (*p* = 0.038) and (*p* = 0.011) for MDA-MB-231, MCF-7 and SKBR-3, respectively
			↓ PON2 expression in BC cells
Campagna et al. (2022)	Oral squamous cell carcinoma	HSC-3 and HOC621	HSC3 cells; both vectors reported ↓ PON2 mRNA with pLKO.1–643 (0.7078 Â ± 0.1576; *p* = 0.0475) and pLKO.1–647 (0.3435 Â ± 0.0013; *p* = 0.0010)
			HOC621 cells ↓ PON2 mRNA levels with pLKO.1–647 (0.5864 Â ± 0.0660; *p* = 0.0352) but not with pLKO.1–643
			HSC3 cells; ↓ PON2 expression with pLKO.1–647 (0.5564 Â ± 0.0301; *p* = 0.0004)
			HOC621 cells; ↓ PON2 expression with pLKO.1–647 (0.4928 Â ± 0.0289; *p* = 0.0003)
Campagna et al. (2020)	Melanoma (skin cancer)	A375	↓ PON2 mRNA level (*p* < 0.05) in pLKO.1647 treated A375 cells (0.24 Â ± 0.01)
			↓ PON2 protein levels (*p* < 0.05) in pLKO.1647 treated cells (2.99 Â ± 0.31)

^†^This table presents data on PON2 expression modulation in response to therapeutic interventions across various cancer models. It includes in vitro and in vivo studies, detailing increases (↑) or decreases (↓) in PON2 expression in different cancer types, cell lines, and animal models. Findings are supported by techniques such as RT−qPCR, Western blot, immunocytochemistry, and gene silencing. The results highlight PON2’s role in tumor progression, metabolic regulation, and therapeutic response, underscoring its potential as a biomarker and therapeutic target in cancer treatment

Abbreviation: T24: bladder cancer cell line, CaCo2: colorectal adenocarcinoma, CSE: *C. spinosa subsp. rupertris*, C12: N−(3−Oxododecanoyl)−homoserine lactone, HeLa: cervical cancer cell lines, A549: lung carcinoma cell line, HCT116: human colorectal carcinoma cell line, NBHE: normal bronchial human epithelial cells, NSCLC: non−small cell lung cancer, BCPAP: human thyroid papillary carcinoma cell line, BHP5−16: human thyroid cancer cell line, TPC−1: thyroid papillary carcinoma cell line, CGTH−W3: human thyroid cancer cell line, EA hy 926: endothelial cells hybrid between human umbilical vein endothelial cells (HUVECs) and A549 cells, HEK293: human embryonic kidney 293 cells, MGC−803, SGC−7901 and BGC−823: human gastric cancer cell line, MKN45 and AGS: human gastric cancer cell line derived from adenocarcinoma, AITL: Angioimmunoblastic T−cell lymphoma, GC: gastric cancer, U87, GBM8401 and DBTRG05MG: glioblastoma−derived cell lines, HepG2: human liver cancer cell line, U87−MG: human malignant glioblastoma cell line, MRC5−V2: normal lung tissue, SKOV3: ovarian cancer cell line, HT1080: human fibrosarcoma cell line, ccRCC−786: human renal cell carcinoma cell line, Caki−1: human kidney cancer cell line, IMR−32: neuroblastoma cell line, B−ALL cell: B−cell acute lymphoblastic leukaemia, LLC: Lewis lung carcinoma, PANC1: human pancreatic cancer cell line derived from ductal carcinoma of pancreas, AsPC−1: human pancreatic cancer cell line derived from metastatic site of pancreatic adenocarcinoma, MiPaCa−2: human pancreatic cancer cell line derived from primary pancreatic tumour, SU.86.86: human pancreatic adenocarcinoma derived from liver metastasis of pancreatic cancer, PDAC: Pancreatic ductal adenocarcinoma, HPNE−hTERT: human pancreatic nestin− expressing cell line derived from ductal carcinoma of pancreas, HPNE−hTERT E6/E7/st: modified version of HPNE−hTERT cell line, SCC−4: human tongue squamous cell carcinoma, PCI−13: human head and neck squamous cell carcinoma cell line, K562: human chronic myelogenous leukemia cell line, HUVEC: human umbilical vein endothelial cells, Ph+: Ph chromosome, MCF7(ER+): human breast cancer cell line (estrogen−positive), SKBR3(HER+): human breast cancer cell line (human epidermal growth factor receptor 2), MDA−MB231: human breast cancer cell line(triple negative breast cancer), HSC3 and HOC621: human oral squamous cell carcinoma cell line, VPA: valproic acid, CFP: chlorpyrifos, PA: papthion, DTIC: dacarbazine, CDDP: cisplatin, 5−FU: 5−fluorouracil, pcDNA3−PON2: PON2 expression vector, DOX: doxorubicin, GBM: glioblastoma multiform, DKO−MEF: double knock−out mouse embryos, MEF: Mouse embryonic fibroblast

Bladder cancer cells (T24) with treatment with the plant *C. spinosa subsp. rupestris* (CSE) induced substantially elevated levels of PON2, especially at lower CSE concentrations (Bacchetti et al. 2022). Significantly increased levels of PON2 were observed in HeLa cells during doxycycline treatment (Ng et al. 2001). PON2 overexpression was observed in tumour tissues, which was in contrast to adjacent normal tissues, while PON2 expression was steadily decreased using shRNA (short hairpin RNA) in the non-small cell lung cancer (NSCLC) cell lines A549 and NCI-H1299 (Zhao et al. 2016). It has been revealed that PON2 amplification in multiple cancer cell lines lowers endoplasmic reticulum stress-induced apoptosis and mitochondrial superoxide generation (Witte et al. 2011), whereas it has been found that miR-376a-3p targets and downregulates PON2 by inflicting post-transcriptional suppression in thyroid cancer cells (Xie et al. 2021). Raised PON2 levels in human lung adenocarcinoma cells, particularly altered cell cycle progression and metabolism, revealing a steady decline of PON2 in Lewis Lung Carcinoma cells through RNA interference (Whitt et al. 2023). Others have shown that si-PON2 transfection decreases PON2 levels inside gastric carcinoma cells while increased PON2 mRNA was detected in gastric cancer (GC) tissues, with elevated levels being associated with a reduced likelihood of survival (Wang et al. 2019). In glioblastoma-derived cell lines, valproic acid (VPA) causes a dose-dependent downregulation of PON2, which is reversible by 1-benzoyl-3-phenyl-2-thiourea (Tseng et al. 2017). HepG2 cells subjected to lead acetate led to a strong decrease in PON2 activity, despite an increase in mRNA levels. The degree of activity loss was restored by the addition of calcium (Sukketsiri et al. 2013). PON2 expression was found to spike in brain and liver malignancies, while leukaemia and glioblastoma display gene loss and amplification, respectively (Shakhparonov et al. 2018). Along similar lines, PON2 was overexpressed in N-3-Oxo-dodecanoyl-L-homoserine lactone (C12)-sensitive mouse embryonic fibroblasts and rescued C12-induced apoptosis based on a mechanism independent of Bax or Bak (Schwarzer et al. 2015). Furthermore, significant reductions in PON2 expression at both the mRNA and protein levels were observed in human renal cell carcinoma lines treated with chemotherapeutic agents such as cisplatin (CDDP) and 5-fluorouracil (5-FU), as well as through shRNA-mediated gene silencing, which also contributed to a decrease in cell proliferation rates. (Schiavoni et al. 2024). A study examining squamous carcinoma cells (SCC) tissue samples showed that tumour tissues had significantly higher levels of PON2 expression than healthy margins, regardless of no discernible correlation with clinical characteristics (Sartini et al. 2021). Another study investigated RHOA G17V mutation which discovered that PON2 expression was found to be elevated through the PI3K/Akt/IKK/NF-κB pathway, while PON2 levels dropped by PI3K inhibition (Que et al. 2022). Neuroblastoma cell lines treated with chlorpyrifos (CPF) contained low PON2 protein levels while exposure to papthion (PA) enhanced PON2, but minimal paraoxonase activity was seen (Parween et al. 2022). A study deployed CRISPR-Cas9 to delete PON2 in B-cell lymphoblastic leukaemia (B-ALL) models, where PON2 loss disrupted cell cycle regulation and delayed cell proliferation and discovered that high PON2 levels had been linked with poor survival in paediatric B-ALL (Pan et al. 2021). PON2 expression has been reported to be influenced by p53 and PON2 knockdown in pancreatic ductal adenocarcinoma cell (PDAC) cell lines inhibits tumour growth and alters glucose metabolism (Nagarajan et al. 2017). In human samples and oral squamous cell carcinoma (OSCC), cell lines exhibited fluctuating PON2 expression, with elevated levels following radiation (Krüger et al. 2015; Kamal et al. 2025). Furthermore, VPA therapies and Wnt ligands (Wnt 3a and 5a) raised PON2 levels in different cell lines, each having an impact on β-catenin activity (Krüger et al. 2016). Dexamethasone (DEX) sensitivity was found to be increased in leukaemia cell lines through PON2 knockdown (Hui et al. 2022). Elevated PON2 levels have been identified in ovarian cancer phases and metastases, confirming its function as a cancer biomarker (Devarajan et al. 2018). Using CDDP and other medications, successfully silenced the PON2 gene in several kinds of cancer cell lines, resulting in notable drops in PON2 mRNA and protein levels (Campagna et al. 2020, 2022, 2023; Belloni et al. 2025). Significant PON2 overexpression was additionally identified in clinical samples from multiple kinds of malignancies as well as bladder cancer cell lines (Bacchetti et al. 2017, 2021). Lastly, overexpression of PON2 decreased oxidative stress and superoxide generation, proving its function in regulating cellular responses to oxidative damage (Altenhöfer et al. 2010). Together, these studies highlight multiple roles and regulatory pathways of PON2 in cancer, emphasising the protein's potential as a diagnostic and therapeutic target.

### The influence of PON2 on cancer cell proliferation and viability

PON2 could serve as an informative biomarker and/or a potential drug target considering its crucial role in regulating cancer cell behaviour in various cancer cell lines and models. Table 3 summarises the involvement of PON2 with numerous examples, some of which are discussed in this section. CSE plant extracts strongly inhibit the proliferation of Caco-2 colorectal cancer cells and T24 bladder cancer cells, with the latter having a particularly robust anti-proliferative effect (Bacchetti et al. 2022). PON2-deficient A549 cells were found to hinder C12-induced apoptosis, unlike cells expressing PON2, highlighting that PON2 is essential for C12's cytotoxic effects (Zhao et al. 2016). PON2 knockdown partially inhibits the proliferation of thyroid cancer cells produced via miR-376a-3p inhibitors revealing that PON2 has a complex regulatory function in these cells (Xie et al. 2021). PON2 deficiency was found to limit lung adenocarcinoma and LLC cell proliferation in vivo and in vitro (Whitt et al. 2023). Furthermore, PON2 knockdown was observed to restrict the viability, migration, and invasion of GC cells, outlining its function in the advancement of cancer (Wang et al. 2019). Through PON2 inhibition, VPA inhibits the growth of glioblastoma growth cells (Tseng et al. 2017). C12 had been found to induce concentration-dependent cell death in HEK293T cells expressing wild-type PON2 (Schwarzer et al. 2015). PON2 downregulation in renal cell carcinoma triggered a drop in cell viability, which became more apparent after CDDP and 5-FU treatment (Schiavoni et al. 2024). In AITL, it has been reported that the presence of the RHOA G17V mutation elevates PON2 production and proliferation, while PON2 knockdown attenuated these effects (Que et al. 2022). In B-ALL, CRISPR-Cas9 deletion of PON2 impairs colony-formation and proliferation (Pan et al. 2021). PON2 also promotes colony formation and metastasis in PDAC, and its knockdown slows the growth of tumours by triggering apoptotic pathways (Nagarajan et al. 2017). In another study, PON2 knockdown in leukaemia cells increased cell sensitivity to DEX and consequently reduced tumour volume (Hui et al. 2022). Consistent reductions in proliferation and improved chemotherapeutic sensitivity have been reported in multiple cancer models following PON2 silencing (Campagna et al. 2020, 2022, 2023). PON2 was observed to be reducing the binding of c-Jun to IGF1 (Insulin-like growth factor 1) promoter, thereby decreasing IGF1 expression and indirectly limits cellular proliferation as a result even in higher PON2 levels, cell proliferation was observed to be regulated (Devarajan et al. 2018). A correlation has also been observed between PON2 overexpression and poor prognosis in melanoma with heightened cell proliferation and tumour malignancy in the bladder and basal cell carcinomas (Bacchetti et al. 2017, 2021). These findings emphasise the critical role that PON2 performs in maintaining proliferation, survival, and responsiveness to the treatment of cancerous cells.

**Table 3:** Influence of PON2 on cancer cell proliferation and viability across various cancer types and models

Study ID	Investigated cancer type	Cell line/animal model	Cancer cell proliferation and viability influenced by PON2
Bacchetti et al. (2022)	Advanced-stage human bladder cancer and human colorectal adenocarcinoma	T24, Caco2, UROtsa and HuDe	CSE exhibited ↑ anti-proliferative effect in Caco-2 and T24 cells vs UROtsa and HuDe cells
			CSE exhibited ↑ anti-proliferative effects particularly in Caco-2 cells, even at the lowest conc
			T24 cells showed ↓ viability at higher CSE conc
Zhao et al. (2016)	–	A549, HCT116, NHBE	C12 inhibited the growth of A549 tumours with vector control but not in tumours with reduced PON2, highlighting the necessity of PON2 for C12's in vivo efficacy
		In vivo model: C57BL/6 mice or in 6-week-old athymic nude mice	
Xie et al. (2021)	Thyroid cancer	BCPAP, BHP5-16, TPC-1 and CGTH-W3	PON2 knockdown partially ↓ proliferation induced by the miR-376a-3p inhibitor
Whitt et al. (2023)	LLC and lung adenocarcinoma cells	A549, NCIH1299, and HEK-293 T cells	HEK-293 T and HBE cells lacking PON2, proliferated at same rate as empty vector
			PON2 expression deficiency hampers lung adenocarcinoma cell proliferation
		In vivo model: KrasLSL-G12D mouse model of primary lung tumorigenesis	Demonstrated that PON2 deficiency in LLC cells reduced their proliferation in vitro
Wang et al. (2019)	GC	MGC-803, AGS, BGC-823, MKN45 and SGC-7901	MTT Assay: PON2 knockdown inhibited GC cell viability at 24, 48, and 72 h (*p* < 0.001)
			Migration Assay: PON2 knockdown ↓ migration of MKN45 and SGC-7901 cells (*p* < 0.001)
			Invasion Assay: PON2 knockdown ↓ invasion of MKN45 and SGC-7901 cells (*p* < 0.001)
Tseng et al. (2017)	GBM	U87, GBM8401, and DBTRG05MG	VPA (at 5, 10, and 20 mM) ↓ cell growth in U87, GBM8401, and DBTRG-05MG GBM cells
			↓ cell growth was observed in a time- and dose-dependent manner from 24 to 72 h
		In vivo model: BALB/c nude mice	MTS and Bromodeoxyuridine (BrdU) assays showed ↓ growth with 10 to 20 mM VPA in U87 cells
			↓ growth with 5 to 20 mM VPA in GBM8401 and DBTRG-05MG cells
			VPA caused cell cycle arrest at the G2/M phase at 24 and 48 h in U87, GBM8401, and DBTRG-05MG cells (via Flow cytometry)
			VPA inhibits PON2 transcriptional activity between positions −400/−1 in GBM cells
Schwarzer et al. (2015)	–	HEK293T cells	Concentration-dependent cell killing by C12 was observed in HEK293T cells with wild-type PON2
		In vivo model: Mouse embryonic fibroblast (MEF)—Wild type, Bax/Bak double knock-out mouse embryos (DKO MEF)	
Schiavoni et al. (2024)	Renal cell carcinoma	ccRCC-786 and Caki-1	MTT without treatment: ↓ cell viability (p < 0.05) in both cell lines due to PON2 downregulation
			786-O and Caki-1 Cells; Steady reduction in cell proliferation at 48 and 72 h
			0.1 µM CDDP ↓ cell viability in PON2-silenced cells and 10 µM CDDP reduced viability in both PON2-silenced vs control. (786-O cells via MTT)
			↓ cell viability for 5-FU conc. (5 and 10 µM) in PON2-silenced cells. (786-O cells via MTT)
			↓ cell viability at all CDDP conc. at 48 and 72 h. (Caki-1 cells via MTT)
			↓ cell viability at all 5-FU conc. at 48 and 72 h. (Caki-1 cells via MTT)
Que et al. (2022)	AITL	54—FFPE tissue samples	Expression of Rhoa G17V in CD4 + T cells, ↑ cell proliferation with ↑ of PON2
			PON2 knockdown ↓ Tfh cell-stimulated germinal center B cells
			PON2 knockdown ↓ proliferation and induces apoptosis in Rhoa G17V-expressing CD4 + T cells, indicating PON2's oncogenic role in Tfh-associated malignancies
Pan et al. (2021)	B-ALL	B-ALL Cells	CRISPR-Cas9-mediated deletion of PON2 in human (Ph chromosome) Ph + B-ALL cells (BV173) reduces colony-forming capacity and proliferation
		In vivo model: PON2—-/- -, mice (26) were backcrossed to wild-type C57BL/6 J for more than 8 generations	
Nagarajan et al. (2017)	PDAC	PANC1, AsPC-1, MiaPaCa-2, SU.86.86, HPNE-hTERT, and HPNE-hTERT E6/E7/st cell line	PON2 ↑ colony-forming ability of KRAS G12D-transformed cells in soft agar and accelerated tumour formation in mice
			PON2 knockdown inhibited lung and liver metastases; ↓ circulating tumour cells vs controls
		In vivo model: Athymic nude mice (both male and female) (NCr nu/nu, 8 weeks)	PON2 is necessary for anoikis resistance, facilitating tumour growth and metastasis in PANC1 and AsPC-1 PDAC cells
		2 – mouse models: a subcutaneous tumour xenograft model and orthotopic pancreatic tumour xenograft model	PON2 knockdown in PDAC cells triggers cellular starvation, activating FOXO3A and PUMA, leading ↓ tumour growth and metastasis
Hui et al. (2022)	ALL, B-ALL and T-ALL	In vivo model: Injected with Human leukaemia cell lines, REH (DEX-resistant) and	Tumour growth ↓ in mice injected with PON2-KD cells
		SUP-B15 (DEX-sensitive)	↓ tumour volume in the combined treatment group (PON2-KD + DEX)
Devarajan et al. (2018)	Ovarian cancer	ID-8, SKOV3, HeLa, A549	BrdU Incorporation assay:
			↓ proliferation in ID8-hPON2 cells vs ID8-EV cells, significant by days 2 and 3
		In vivo model: Mouse xenograft model of ovarian cancer	↑ proliferation in PON2-siRNA transfected SKOV3 cells, but not in HeLa and A549 cells
Campagna et al. (2023)	Triple-negative breast cancer	MCF-7 (ER +), SKBR-3 (HER2 +) and MDA-MB-231 (TNBC)	MTT Assay:
			↓ proliferation observed starting at 48 h (p = 0.039) and more evident at 72 h (*p* = 0.022) (in MDA-MB-23 cells)
			↓ proliferation at 72 h (*p* = 0.036) vs control and empty vector-transfected cells. (in MCF-7 and SKBR-3 cells)
			MTT Assay after treatment MDA-MB-231 cells: (↓ cell viability)
			DOX: 0.1uM at 24 h (*p* = 0.032) and 48 h (*p* = 0.032) and 72 h (*p* = 0.032); 1uM at 24 h (*p* = 0.042) and 48 h (*p* = 0.032); 10uM at 24 h (*p* = 0.019)
			5-FU: 0.5uM at 24 h (*p* = 0.028) and 48 h (*p* = 0.037) and 72 h (*p* = 0.025); 1uM at 72 h (*p* = 0.043);
			5uM at 24 h (*p* = 0.033) and 48 h (*p* = 0.036)
			CDDP: 1uM at 24 h (*p* = 0.026) and 48 h (*p* = 0.030)
			Combination Treatment: 10 nM DOX + 100 nM CDDP + 50 ng/ml 5-FU; ↓cell viability at 48 h (*p* = 0.037) and 72 h (*p* = 0.039)
Campagna et al. (2022)	Oral squamous cell carcinoma	HSC-3 and HOC621	PON2 silencing ↓ cell viability at 72 h in OSCC cell lines HSC-3 and HOC621 (via MTT)
			PON2 silencing results in ↓ cell proliferation (via Trypan Blue assay)
			After CDDP treatment on PON2 silenced cell lines:
			0.25 µg/mL CDDP significantly (*p* < 0.0001) ↓ cell viability in PON2 silenced HSC cells (pLKO.1–647) at 96 h
			↓ cell viability at 48 h after treatment with 1 µg/mL CDDP (*p* < 0.0001) in PON2 silenced HOC621 cells (pLKO.1–647)
Campagna et al. (2020)	Melanoma (skin cancer)	A375	↓ Cell proliferation in PON2 silenced cells at (*p* < 0.05) along with ↓ cell viability (*p* < 0.05) at 48 h and 72 h
			PON2 silencing had no significant effect on A375 cell sensitivity to DTIC
			PON2 silencing ↑ sensitivity to 4 µM CDDP (*p* < 0.05), while 8 µM CDDP causes excessive cell viability ↓ regardless of PON2 status
Bacchetti et al. (2017)	Bladder cancer	T24	↑ cell growth at 72 h (*p* < 0.05) due to PON2 upregulation (via MTT)
			↑ T24 cell proliferation, suggesting a role in promoting bladder tumorigenesis
			↑ cell proliferation marker MIB-1 (Ki-67) (*p* < 0.05) in PON2 overexpressing cells (3.92 ± 0.13) (via RT-PCR)
			Upregulation of Ki-67 ↑ proliferative capacity in PON2 overexpressing cells
			(Ki-67 expressed in in proliferating cells (G1, S, G2, and mitosis phases)
Bacchetti et al. (2021)	Basal cell carcinoma and melanoma	FFPE tissue specimens	↑ PON2 expression correlates with tumour aggressiveness for BCC
			↑ PON2 expression is correlated with unfavourable prognosis in melanoma patients

^†^This table presents an overview of the influence of PON2 on cancer cell proliferation and viability across various cancer types and experimental models. It includes both in vitro studies using diverse cancer cell lines and in vivo research utilizing mouse models. The data are derived from various assays, including MTT, BrdU incorporation, colony formation, migration, invasion, and flow cytometry. These findings illustrate PON2's significant role in tumor progression, therapeutic resistance, and metastasis, highlighting its potential as both a biomarker and a therapeutic target in cancer treatment

Abbreviation: T24: bladder cancer cell line, CaCo2: colorectal adenocarcinoma, UROtsa: Immortalized human urothelial cell line, HuDe: human dermal fibroblast, CSE: *C. spinosa subsp. rupertris*, C12: N−(3−Oxododecanoyl)−homoserine lactone, HeLa: cervical cancer cell lines, A549: lung carcinoma cell line, HCT116: human colorectal carcinoma cell line, NBHE: normal bronchial human epithelial cells, NSCLC: non−small cell lung cancer, BCPAP: human thyroid papillary carcinoma cell line, BHP5−16: human thyroid cancer cell line, TPC−1: thyroid papillary carcinoma cell line, CGTH−W3: human thyroid cancer cell line, EA hy 926: endothelial cells hybrid between human umbilical vein endothelial cells (HUVECs) and A549 cells, HEK293: human embryonic kidney 293 cells, MGC−803, SGC−7901 and BGC−823: human gastric cancer cell line, MKN45 and AGS: human gastric cancer cell line derived from adenocarcinoma, U87, GBM8401 and DBTRG05MG: glioblastoma−derived cell lines, HepG2: human liver cancer cell line, U87−MG: human malignant glioblastoma cell line, MRC5−V2: normal lung tissue, SKOV3: ovarian cancer cell line, HT1080: human fibrosarcoma cell line, ccRCC−786: human renal cell carcinoma cell line, Caki−1: human kidney cancer cell line, IMR−32: neuroblastoma cell line, B−ALL cell: B−cell acute lymphoblastic leukaemia, PANC1: human pancreatic cancer cell line derived from ductal carcinoma of pancreas, AsPC−1: human pancreatic cancer cell line derived from metastatic site of pancreatic adenocarcinoma, MiPaCa−2: human pancreatic cancer cell line derived from primary pancreatic tumour, SU.86.86: human pancreatic adenocarcinoma derived from liver metastasis of pancreatic cancer, HPNE−hTERT: human pancreatic nestin− expressing cell line derived from ductal carcinoma of pancreas, HPNE−hTERT E6/E7/st: modified version of HPNE−hTERT cell line, SCC−4: human tongue squamous cell carcinoma, PCI−13: human head and neck squamous cell carcinoma cell line, K562: human chronic myelogenous leukemia cell line, HUVEC: human umbilical vein endothelial cells, MCF7(ER+): human breast cancer cell line (estrogen−positive), SKBR3(HER+): human breast cancer cell line (human epidermal growth factor receptor 2), MDA−MB231: human breast cancer cell line(triple negative breast cancer), HSC3 and HOC621: human oral squamous cell carcinoma cell line, VPA: valproic acid, CFP: chlorpyrifos, PA: papthion, DTIC: dacarbazine, CDDP: cisplatin, DEX: dexamethasone, 5−FU: 5−fluorouracil, pcDNA3−PON2: PON2 expression vector, FFPE: 92 formalin−fixed and paraffin−embedded, AITL: Angioimmunoblastic T−cell lymphoma, LLC: Lewis lung carcinoma, PDAC: Pancreatic ductal adenocarcinoma, ALL: acute lymphoblastic leukemia, T−ALL: T−cell lymphoblastic leukemia, DKO−MEF: double knock−out mouse embryos, MEF: Mouse embryonic fibroblast, GBM: glioblastoma multiform, GC: gastric cancer

### PON2 modulation of ROS and oxidative stress in cancer

Table 4 indicates the significance of PON2 in managing oxidative stress and ROS in a range of cancer models and types. In T24 bladder cancer and Caco2 colorectal cancer cells, treatment with extracts of the CSE plant raised ROS levels. Furthermore, there was a correlation between enhanced PON2 levels and ROS induction (Bacchetti et al. 2022). A significant PON2 overexpression in HeLa cervical cancer cells alleviates the oxidative damage induced by hydrogen peroxide (Ng et al. 2001). Similarly, enhanced intracellular ROS levels were observed as a direct consequence of the lack of PON2 in A549 lung cancer cells (Zhao et al. 2016). This was further supported by experiments assessing the effect of PON2 on CHOP protein levels using PON2 point mutations H114Q and H113Q, which lack lactonase activity but retain antioxidative function. The observed reduction in CHOP levels suggests that PON2’s antioxidative ability plays a key role in protecting cells from elevated ROS generation and apoptosis. (Witte et al. 2011). VPA treatment elevated ROS levels in glioblastoma cells, particularly within PON2-silenced cells, however, PON2 overexpression significantly reduced ROS production (Tseng et al. 2017). In HepG2 hepatoma cells, lead acetate-induced oxidative stress has been associated with reduced PON2 activity, however this could potentially be restored with calcium treatment (Sukketsiri et al. 2013). PON2 has been shown to lower the production of ROS within mitochondria and shield cell membranes against oxidative damage in various cancer cell lines (Shakhparonov et al. 2018). It has been observed that the chemical C12 triggered ROS production and mitochondrial depolarisation, particularly in PON2-expressing cells, which prompted apoptosis (Schwarzer et al. 2015). PON2 downregulation in renal cell cancer cells impaired antioxidant defences reflected by elevated ROS levels upon CDDP and 5-FU treatment (Schiavoni et al. 2024). PA and CPF exposure have been observed to elevate the production of ROS in neuroblastoma cells, however, PA has been also found to elevate PON2 levels which ensure proper oxidative stress regulation (Parween et al. 2022). PON2 overexpression appears to improve the activity of the mitochondrial electron transport chain and lower the level of superoxides in ovarian cancer cells (Devarajan et al. 2018). PON2 gene silencing in melanoma cells has been reported to stimulate ROS production, especially when treated with CDDP (Campagna et al. 2020). Temporary overexpression of PON2 in bladder cancer cells suppressed ROS formation after treatment with tert-butyl hydroperoxide (TBHP) (Bacchetti et al. 2017). Lastly, a research study noted that PON2 mutations in lung cancer cells reduced their antioxidative activity, revealing a crucial role of PON2 in preventing mitochondrial apoptosis (Altenhöfer et al. 2010). These studies suggest that PON2 may be a useful target in oxidative stress-related cancer progression.

**Table 4:** Modulation of ROS and oxidative stress by PON2 in various cancer types

Study ID	Investigated cancer type	Cell line/animal model	ROS/oxidative stress impact by PON2 expression in cancer
Bacchetti et al. (2022)	Advanced-stage human bladder cancer and human colorectal adenocarcinoma	T24, Caco2, UROtsa and HuDe	CSE-induced ROS was tested at 48 h:
			56% ↑ ROS at 10ug GAE/ml; (p < 0.001) (in Caco2)
			24% ↑ ROS at 50ug GAE/ml; (p < 0.005) (in T24)
			↑ PON2 levels in T24 cells with (p < 0.001); Induced ROS by CSE vs ↓ PON2 levels in control
Ng et al. (2006)	Cervical cancer	HeLa cell lines	Hydrogen peroxide treatment ↓ doxycycline-induced HeLa-Tet-PON2 cells that overexpressed PON2 protein vs untreated controls (via Fluorescence spectroscopy)
Zhao et al. (2016)	–	A549, HCT116, NHBE	↓ PON2 expression; ↑ intracellular ROS in A549 and NCI-H1299 cells
		In vivo model: C57BL/6 mice or in 6-week-old athymic nude mice	
Witte et al. (2011)	Leukaemia and lung carcinoma	EA hy 926 cells, HEK293, K562, A549	Mutations (H114Q or H133Q) indicate that PON2’s anti-apoptotic function is based on its anti-oxidative effect
			PON2 knockdown induces ↑ ROS production and moderate CHOP expression
			Mitochondrial superoxide accumulation was ↓ PON2-overexpressing cells
			Isolated mitochondria from PON2-overexpressing cells produced ↓ superoxide vs controls
Tseng et al. (2017)	GBM	U87, GBM8401, and DBTRG05MG	10 mM VPA treatment ↑ ROS levels in U87 cells for 24 to 48 h
			5 mM VPA treatment ↑ ROS levels in GBM8401 and DBTRG-05MG cells for 24 to 48 h
		In vivo model: BALB/c nude mice	↑ PON2-silenced cells in the presence of VPA vs controls
			ROS level ↓ PON2-overexpressed cells stimulated with VPA vs controls in U87 and GBM8401
Sukketsiri et al. (2013)	Hepatoma	HepG2 cells	Lead acetate induces oxidative stress; ↓ PON2 activity in HepG2 cells
			Restoration of PON2 activity by Ca^2+^, after 30 min incubation of HepG2 in lead acetate followed by incubation with CaCl_2_ for another 30 min
			HepG2 cells respond to lead-induced oxidative stress and ↓ PON2 activity by ↑ PON2 expression
Shakhparonov et al. (2018)	Liver carcinoma, glioblastoma, lung cancer, ovarian carcinoma, lung carcinoma, leukaemia, fibrosarcoma	U87-MG, MRC5-V2, SKOV3, A549,	PON2 functions include lactone cleavage, ↓ mitochondrial ROS production, and protection of membrane lipids against peroxidation
		HepG2 and, HT1080	PON2 protects intracellular membranes from oxidation
			Prevents free radicals from damaging genetic material within cells
Schwarzer et al. (2015)	–	HEK293T cells	C12 caused significant, sustained mitochondrial depolarization in hPON2-expressing DKONR MEF cells
			HEK293T cells (wild-type PON2), C12 led to maximal mitochondrial depolarization (86%)
		In vivo model: Mouse embryonic fibroblast (MEF)—Wild type, Bax/Bak double knock-out mouse embryos (DKO MEF)	PON2's in ER and mitochondria indicates C12 cleaves PON2, generate ROS triggering apoptosis
Schiavoni et al. (2024)	Renal cell carcinoma	ccRCC-786 and Caki-1	↑ PON2 downregulates cells vs controls, suggesting an impaired antioxidant capacity
			1 µM CDDP; PON2 downregulates up to 2.16 ± 0.156 fold
			5 µM and 10 µM 5-FU; PON2 downregulates up to 2.17 ± 0.325 fold and 1.26 ± 0.147 fold, respectively
Parween et al. (2022)	Neuroblastoma	IMR-32	100 µM CPF exposure for 24 h ↑ ROS generation in IMR-32 cells
			100 µM PA-induced ↑ ROS in IMR-32 cells
			↑ aryl-esterase activity did not enhance the antioxidant property of PON2 in CPF-treated cells
			PA exposure ↑ PON2 protein expression, effectively scavenging ROS, resulting in ↓ ROS levels
Devarajan et al. (2018)	Ovarian cancer	ID-8, SKOV3, HeLa, A549	Mitochondrial ETC Complex II + III activity ↑ ID8-hPON2 cell
		In vivo model: Mouse xenograft model of ovarian cancer	Mitochondrial superoxide levels ↓ ID8-hPON2 cells
Campagna et al. (2020)	Melanoma	A375	↑ ROS production in PON2 downregulating cells (pLKO.1–647) (*p* < 0.05) at 48 h and 72 h
	(skin cancer)		PON2 knockdown ↑ ROS production in response to CDDP treatment in A375 cells
Bacchetti et al. (2017)	Bladder cancer	T24	TBHP treatment for 3 h, ↓ ROS production (*p* < 0.05) in PON2 over-expressing cells vs control
Altenhöfer et al. (2010)	Lung carcinoma	EA.hy 926	O2.-, benefit from PON2's antioxidative properties in preventing apoptosis (in mitochondria)

^†^This table presents data on the modulation of reactive oxygen species (ROS) and oxidative stress by PON2 expression in various cancer types and models. The studies included encompass both in vitro and in vivo experiments, investigating how PON2 affects ROS production, mitochondrial function, and cellular antioxidant defenses. Overall, the data indicate that PON2 plays a protective role in mitigating oxidative stress and ROS−induced damage, thereby influencing tumor progression and responses to treatment. These findings underscore the potential of targeting PON2 as a therapeutic strategy in cancer, particularly for its role in regulating oxidative stress in cancer cells

Abbreviation: T24: bladder cancer cell line, UROtsa: Immortalized human urothelial cell line, HuDe: human dermal fibroblast, CSE: *C. spinosa subsp. rupertris*, C12: N−(3−Oxododecanoyl)−homoserine lactone CaCo2: colorectal adenocarcinoma, CSE: *C. spinosa subsp. rupertris*, C12: N−(3−Oxododecanoyl)−homoserine lactone, HeLa: cervical cancer cell lines, A549: lung carcinoma cell line, HCT116: human colorectal carcinoma cell line, NBHE: normal bronchial human epithelial cells, NSCLC: non−small cell lung cancer, BCPAP: human thyroid papillary carcinoma cell line, BHP5−16: human thyroid cancer cell line, TPC−1: thyroid papillary carcinoma cell line, CGTH−W3: human thyroid cancer cell line, EA hy 926: endothelial cells hybrid between human umbilical vein endothelial cells (HUVECs) and A549 cells, HEK293: human embryonic kidney 293 cells, MGC−803, SGC−7901 and BGC−823: human gastric cancer cell line, MKN45 and AGS: human gastric cancer cell line derived from adenocarcinoma, U87, GBM8401 and DBTRG05MG: glioblastoma−derived cell lines, HepG2: human liver cancer cell line, U87−MG: human malignant glioblastoma cell line, MRC5−V2: normal lung tissue, SKOV3: ovarian cancer cell line, HT1080: human fibrosarcoma cell line, ccRCC−786: human renal cell carcinoma cell line, Caki−1: human kidney cancer cell line, IMR−32: neuroblastoma cell line, B−ALL cell: B−cell lymphoma, PANC1: human pancreatic cancer cell line derived from ductal carcinoma of pancreas, AsPC−1: human pancreatic cancer cell line derived from metastatic site of pancreatic adenocarcinoma, MiPaCa−2: human pancreatic cancer cell line derived from primary pancreatic tumour, SU.86.86: human pancreatic adenocarcinoma derived from liver metastasis of pancreatic cancer, HPNE−hTERT: human pancreatic nestin− expressing cell line derived from ductal carcinoma of pancreas, HPNE−hTERT E6/E7/st: modified version of HPNE−hTERT cell line, SCC−4: human tongue squamous cell carcinoma, PCI−13: human head and neck squamous cell carcinoma cell line, K562: human chronic myelogenous leukemia cell line, HUVEC: human umbilical vein endothelial cells, MCF7(ER+): human breast cancer cell line (estrogen−positive), SKBR3(HER+): human breast cancer cell line (human epidermal growth factor receptor 2), MDA−MB231: human breast cancer cell line(triple negative breast cancer), HSC3 and HOC621: human oral squamous cell carcinoma cell line, VPA: valproic acid, CFP: chlorpyrifos, PA: papthion, DTIC: dacarbazine, CDDP: cisplatin, DEX: dexamethasone, 5−FU: 5−fluorouracil, pcDNA3−PON2: PON2 expression vector, FFPE: 92 formalin−fixed and paraffin−embedded, AITL: Angioimmunoblastic T−cell lymphoma, LLC: Lewis lung carcinoma, PDAC: Pancreatic ductal adenocarcinoma, ALL: acute lymphoblastic leukemia, T−ALL: T−cell lymphoblastic leukemia

### Effects of PON2 on apoptosis and associated mechanisms in cancer

PON2 also impacts apoptosis and its associated mechanisms in multiple forms of cancer (Table 5). Enhanced PON2 and increased ROS in T24 bladder cancer cells treated with CSE may be operating as a defence mechanism against CSE-induced apoptosis (Bacchetti et al. 2022). In A549 lung cancer cells, a PON2 deficit resulted in reduced cell death and caspase-3/7 activation upon C12 treatment, while in PON2-deficient cells, actinomycin D and tunicamycin exposure further bolstered cell mortality (Zhao et al. 2016). A research study on PON2 knockdown was found to mimic the inhibition of apoptosis in thyroid cancer cells mediated via the miR-376a-3p inhibitor (Xie et al. 2021). Elevated PON2 levels in K562 leukaemia cells appeared to prevent imatinib-induced cell death. Additionally, PON2 overexpression in endothelial cells reduced doxorubicin-triggered caspase-3 activation and ATP depletion. Contrastingly, RNAi-mediated PON2 knockdown was found to further elevate the rates of apoptosis and imatinib susceptibility (Witte et al. 2011) while PON2-deficient lung adenocarcinoma cells caused concentration-dependent surges in cell death and activation of caspase-3/7 upon C12 treatment (Whitt et al. 2023).

**Table 5:** PON2’s role in apoptosis regulation and mechanistic pathways in cancer

Study ID	Investigated cancer type	Cell line/animal model	Apoptosis and related mechanisms modulated by PON2 in cancer
Bacchetti et al. (2022)	Advanced-stage human bladder cancer and human colorectal adenocarcinoma	T24 and Caco2	↑ PON2 in T24 cells treated with CSE-linked ↑ ROS may be a protective response by tumour cells against CSE-induced apoptosis
Zhao et al. (2016)	–	A549, HCT116, NHBE	Cell death and caspase-3/7 activation in PON2-deficient cells treated with C12
		In vivo model: C57BL/6 mice or in 6-week-old athymic nude mice	↑ Cell death and caspase-3/7 activation in PON2-deficient cells treated with actinomycin D and tunicamycin
			↓ cell death and caspase-3/7 activation in HCT116-WT and HCT116-Bak/Bax-DKO cells lacking PON2
Xie et al. (2021)	Thyroid cancer	BCPAP, BHP5-16, TPC-1 and CGTH-W3	PON2 knockdown replicated the miR-376a-3p inhibitor-induced suppression of cell apoptosis in LINC00488-deficient BCPAP cells
Witte et al. (2011)	Leukaemia and lung carcinoma	EA hy 926 cells, HEK293, K562, A549	PON2 overexpression in endothelial cells ↓ DOX-triggered caspase-3 activation and ↓ ATP
			↑ PON2 levels in K562 leukaemia cells conferred resistance to imatinib-induced cell death
			RNAi-mediated PON2 knockdown ↑ susceptibility of K562 cells to imatinib and ↑ apoptosis rates
			PON2 knockdown ↑ cell death in K562, A549, and EA.hy 926 cells
			PON2 overexpressing cells showed ↓ activation of caspases 8, 9, and 3 in response to staurosporine and actinomycin D treatments
Whitt et al. (2023)	LLC and lung adenocarcinoma cells	A549, NCIH1299, and HEK-293 T cells	Control-shRNA LLC cells showed conc. dependent ↑ cell death and caspase-3/7 activation upon C12 exposure
		In vivo model: KrasLSL-G12D mouse model of primary lung tumorigenesis	
Tseng et al. (2017)	GBM	U87, GBM8401, and DBTRG05MG	VPA ↓ migration and invasion abilities of U87, GBM8401, and DBTRG-05MG cells
			↑ sub-G1 phase by VPA treatment, indicating ↑ apoptosis in U87, GBM8401, and DBTRG-05MG cells (via PI staining and flow cytometry)
		In vivo model: BALB/c nude mice	Proliferation and apoptotic-related molecules influenced by VPA included:
			↑: Bim, p21
			↓: Cyclin B1, cdc2, Bcl-xL
			VPA regulates PON2, p27, Bim, p21, cyclin B1, cdc2, and Bcl-xL to influence GBM cell proliferation and apoptosis
Schwarzer et al. (2015)	–	HEK293T cells	↑ activation of caspase 3/7 in hPON2-expressing DKONR MEF
			C12 induced ↑ activation of caspase 3/7 in HEK293T cells expressing wild-type PON2
		In vivo model: MEF—Wild type, Bax/Bak DKO MEF	C12 induces pro-apoptotic responses including caspase 3/7 activation, cytochrome c release from mitochondria, and cell killing in cells expressing ↑ PON2 (WT and DKOR MEF)
			Cells with ↓ PON2 expression (DKONR MEF) show no response to C12
			Lactonase activity of PON2, rather than protecting cells from apoptosis, facilitates C12-induced apoptotic responses
Schiavoni et al. (2024)	Renal cell carcinoma	ccRCC-786 and Caki-1	MTT without treatment:
			↓ Ki-67; (0.56 ± 0.112 fold) due to PON2 silencing
			↑ Caspase-3; (1.99 ± 0.187 fold) due to PON2 silencing
			MTT with treatment:
			↓ Ki-67 in PON2-silenced cells treated with CDDP (1 µM: 0.66 ± 0.140 fold) and 5-FU (5 µM: 0.60 ± 0.148 fold; 10 µM: 0.43 ± 0.097 fold)
			↑ Caspase-3 in PON2-silenced cells treated with CDDP (1 µM: 1.80 ± 0.258 fold) and 5-FU (5 µM: 1.29 ± 0.204 fold; 10 µM: 1.49 ± 0.123 fold)
Que et al. (2022)	AITL	54—FFPE tissue samples	PON2 knockdown in Rhoa G17V-expressing CD4 + T cells ↓ proliferation and ↑ apoptosis
			PON2-positive tumours ↓ overall survival than those with PON2-negative tumours (*P* = 0.019)
Pan et al. (2021)	B-ALL	B-ALL Cells	PON2 hydrolyses the bacterial signalling molecule lactone-3OC12 to cytotoxic 3OC12 acid, inducing intracellular acidification and caspase 3/7-dependent apoptosis
		In vivo model: PON2 ^− −^/^− −^, mice (26) were backcrossed to wild- type C57BL/6 J for more than 8 generation	3OC12 treatment induces ↑ phosphorylation of proapoptotic p38 MAPK and activates caspase-3 in patient-derived B-ALL cells with ↓ PON2 expression
Nagarajan et al. (2017)	PDAC	PANC1, AsPC-1, MiaPaCa-2, SU.86.86, HPNE-hTERT, and HPNE-hTERT E6/E7/st cell line	PON2 knockdown induced anoikis (programmed cell death due to detachment)
			Detached PDAC cells with PON2 knockdown; ↑ annexin V-positive cells and ↑ PARP cleavage, ↑ gene encoding PUMA indicating apoptosis
		In vivo model: Athymic nude mice (both male and female) (NCr nu/nu, 8 weeks old)	PON2 knockdown ↓ ATP/ADP and ADP/AMP ratios, indicating cellular starvation
		2 – mouse models: a subcutaneous tumour xenograft model and an orthotopic pancreatic tumour xenograft model	
Krüger et al. (2015)	Oral squamous cell carcinoma	PCI-13, PCI-52, SCC-4, SCC-68	↑ caspase 3/7 activity (via RNAi-mediated PON2 knockdown)
			PCI-13: 7.7 ± 0.12 (48 h), 3.1 ± 0.27 (72 h)
			PCI-52: 3.1 ± 0.27 (72 h)
			SCC-4: 1.5 ± 0.13 (48 h), 1.6 ± 0.25 (72 h)
			SCC-68: 5 ± 0.24 (72 h)
			PON2 knockdown ↑ cell death rates in OSCC cells (via western blot)
Krüger et al. (2016)	Oral squamous cell cancer and leukaemia	SCC-4 and PCI-13, K562, HUVEC and EA.hy 926 cells	↑ PON2 expression in A549 cells led to apoptosis after PON2 knockdown
			OSCC cell lines, PON2 deficiency ↑ susceptibility to irradiation-triggered cell death
Hui et al. (2022)	ALL, B-ALL and T-ALL	In vivo models: Human leukaemia cell lines, REH (DEX-resistant) and	PON2 silencing ↑ apoptosis in SUP-B15R and REH cells treated with DEX (1 μM for 24 h)
		SUP-B15 (DEX-sensitive)	↑ caspase-3 activity in PON2-silenced REH and SUP-B15R cells after DEX treatment
			DEX treatment:
			↓ Bcl-2 mRNA and protein expression in PON2 silenced cells
			↑ Bax mRNA level and protein expression in PON2 silenced cells
			↑ Bax resulted in ↑ apoptotic cells in the combined treatment group (via TUNEL analysis)
Altenhöfer et al. (2010)	Lung carcinoma	EA.hy 926 cells	↓ caspase 3/7 activation was lowest in PON2-H114Q (p < 0.001) vs control treated with tunicamycin (1 μg/ml for 16 h)
			↑ apoptosis was lowest in PON2-H114Q (*p* < 0.01) vs control treated with staurosporine (1 μM, 16 h)

^†^This table outlines the involvement of PON2 in regulating apoptosis and related mechanisms across various cancer types. The studies presented demonstrate how PON2 influences apoptotic pathways, including caspase activation, mitochondrial dysfunction, and resistance to cell death. Both in vitro and in vivo models are considered, illustrating that PON2’s effect on apoptosis can be context−dependent, either promoting or inhibiting cell death in response to factors such as chemotherapy, oxidative stress, or genetic modifications. These findings underscore the potential of PON2 as a therapeutic target in cancer treatment, with its role varying across different cancer types and treatment conditions

Abbreviation: T24: bladder cancer cell line, CaCo2: colorectal adenocarcinoma, CSE: *C. spinosa subsp. rupertris*, C12: N−(3−Oxododecanoyl)−homoserine lactone, HeLa: cervical cancer cell lines, A549: lung carcinoma cell line, HCT116: human colorectal carcinoma cell line, NBHE: normal bronchial human epithelial cells, NSCLC: non−small cell lung cancer, BCPAP: human thyroid papillary carcinoma cell line, BHP5−16: human thyroid cancer cell line, TPC−1: thyroid papillary carcinoma cell line, CGTH−W3: human thyroid cancer cell line, EA hy 926: endothelial cells hybrid between human umbilical vein endothelial cells (HUVECs) and A549 cells, HEK293: human embryonic kidney 293 cells, MGC−803, SGC−7901 and BGC−823: human gastric cancer cell line, MKN45 and AGS: human gastric cancer cell line derived from adenocarcinoma, AITL: Angioimmunoblastic T−cell lymphoma, GC: gastric cancer, U87, GBM8401 and DBTRG05MG: glioblastoma−derived cell lines, HepG2: human liver cancer cell line, U87−MG: human malignant glioblastoma cell line, MRC5−V2: normal lung tissue, SKOV3: ovarian cancer cell line, HT1080: human fibrosarcoma cell line, ccRCC−786: human renal cell carcinoma cell line, Caki−1: human kidney cancer cell line, IMR−32: neuroblastoma cell line, B−ALL cell: B−cell lymphoma, LLC: Lewis lung carcinoma, PANC1: human pancreatic cancer cell line derived from ductal carcinoma of pancreas, AsPC−1: human pancreatic cancer cell line derived from metastatic site of pancreatic adenocarcinoma, MiPaCa−2: human pancreatic cancer cell line derived from primary pancreatic tumour, SU.86.86: human pancreatic adenocarcinoma derived from liver metastasis of pancreatic cancer, PDAC: Pancreatic ductal adenocarcinoma, HPNE−hTERT: human pancreatic nestin−expressing cell line derived from ductal carcinoma of pancreas, HPNE−hTERT E6/E7/st: modified version of HPNE−hTERT cell line, SCC−4: human tongue squamous cell carcinoma, PCI−13: human head and neck squamous cell carcinoma cell line, K562: human chronic myelogenous leukemia cell line, HUVEC: human umbilical vein endothelial cells, Ph+: Ph chromosome, MCF7(ER+): human breast cancer cell line (estrogen−positive), SKBR3(HER+): human breast cancer cell line (human epidermal growth factor receptor 2), MDA−MB231: human breast cancer cell line(triple negative breast cancer), HSC3 and HOC621: human oral squamous cell carcinoma cell line, VPA: valproic acid, CFP: chlorpyrifos, PA: papthion, DTIC: dacarbazine, CDDP: cisplatin, 5−FU: 5−fluorouracil, pcDNA3−PON2: PON2 expression vector, DOX: doxorubicin, GBM: glioblastoma multiform, DKO−MEF: double knock−out mouse embryos, MEF: Mouse embryonic fibroblast, SCC4 and SCC68: Squamous Cell Carcinoma cell line, PCL13 and PCL52: Pittsburgh cancer institute squamous cancer cell clines

VPA treatment may promote apoptosis in glioblastoma cells by modulating critical proteins such as Bcl-xL, p21, Bim, and cyclin B1. Further, it suppresses migration and invasion in these cells (Tseng et al. 2017). PON2 expression amplified C12-induced apoptotic responses, including cytochrome c release and caspase-3/7 activation (Schwarzer et al. 2015). PON2 suppression in renal cell carcinoma cells was found to be contributing to apoptosis by lowering Ki-67 expression and increasing caspase-3 activation (Schiavoni et al. 2024). PON2 knockdown in AITL models reduced proliferation and increased apoptosis, whereas tumours deemed PON2-positive showed poorer survival rates (Que et al. 2022). PON2-induced patient-derived B-ALL cells underwent apoptosis by hydrolysing the bacterial signalling molecule lactone-3OC12 into a potentially cytotoxic acid (Pan et al. 2021). Knocking down PON2 in PDAC cells led to a significant increase in apoptosis, as demonstrated by a higher proportion of annexin V-positive cells and elevated expression of the PUMA gene (Nagarajan et al. 2017). PON2 knockdown strengthened caspase 3/7 activity and cell death in leukaemia and OSCC cells, particularly after irradiation (Krüger et al. 2015, 2016). In ALL cells, PON2 silencing promotes caspase-3 activity and apoptosis following DEX treatment, complemented by a drop in Bcl-2 and an upsurge in Bax expression (Hui et al. 2022). Specific point mutations in PON2 weakened caspase 3/7 activation and apoptosis, indicating that these mutations compromise PON2's ability to block apoptosis (Altenhöfer et al. 2010). Overall, these research findings highlight PON2's complex function in managing cancer cell apoptosis and underline its potential as a therapeutic target for improving cancer cell's sensitivity to treatment-induced apoptosis.

## Discussion

PON2 has emerged as a multifunctional enzyme with profound implications in cancer biology, influencing tumour initiation, progression, therapeutic responsiveness, and resistance. A growing body of evidence highlights its dynamic regulation across various cancer models, where PON2 acts as a key modulator of proliferation, redox balance, and apoptosis. Collectively, these findings underscore the potential of PON2 as both a diagnostic biomarker and a therapeutic target.

Recent studies have firmly established that PON2 is overexpressed in a variety of malignancies, including bladder, gastric, lung, pancreatic, ovarian, and oral cancers, as well as melanoma and leukaemia (Zhao et al. 2016; Bacchetti et al. 2017, 2021; Devarajan et al. 2018; Kamal et al. 2025; Belloni et al. 2025). Elevated PON2 expression correlates with tumour survival, enhanced cell migration, and metastatic potential, often contrasting with its reduced levels in adjacent non-tumour tissues. Functional assays show that PON2 silencing, particularly via shRNA or CRISPR-Cas9, reduces proliferation, induces apoptosis, and sensitises tumour cells to chemotherapeutic agents (Wang et al. 2019; Pan et al. 2021). Mechanistically, PON2 modulates endoplasmic reticulum stress and mitochondrial superoxide generation, thereby protecting tumour cells from oxidative and apoptotic damage (Witte et al. 2011). This anti-apoptotic property is particularly significant in cancers that thrive under elevated oxidative stress, such as lung adenocarcinoma and glioblastoma. Moreover, PON2 expression is regulated by oncogenic signalling pathways, including the PI3K/Akt/IKK/NF-κB axis (Que et al. 2022), and tumour suppressors like p53 (Nagarajan et al. 2017). These findings illustrate that PON2 acts at the intersection of stress adaptation, redox control, and metabolic regulation, making it a central player in cancer pathophysiology.

PON2 also exerts a pronounced influence on cell proliferation and therapeutic response. Its silencing in lung adenocarcinoma, gastric carcinoma, thyroid, and PDAC models consistently leads to reduced proliferation and impaired colony formation (Nagarajan et al. 2017; Xie et al. 2021; Whitt et al. 2023). In leukaemia and B-ALL, PON2 deletion via CRISPR-Cas9 attenuates tumour growth and delays cell cycle progression (Pan et al. 2021; Hui et al. 2022). PON2’s ability to confer chemoresistance is particularly evident in renal cell carcinoma and melanoma, where its downregulation sensitizes cells to CDDP and 5-FU (Campagna et al. 2020; Schiavoni et al. 2024). VPA has also been shown to inhibit glioblastoma growth partly through PON2 suppression (Tseng et al. 2017). Conversely, overexpression of PON2 is associated with aggressive phenotypes and poor prognosis in melanoma, bladder carcinoma, and basal cell carcinoma (Bacchetti et al. 2017, 2021). Interestingly, PON2 can modulate c-Jun binding to the IGF1 promoter, indirectly controlling proliferation even under high PON2 expression levels (Devarajan et al. 2018). These findings suggest that PON2’s proliferative effects are context-dependent and may involve crosstalk with growth factor signalling and transcriptional networks. As such, PON2 inhibition has emerged as an attractive therapeutic strategy, with studies employing RNA interference, small-molecule inhibitors, or plant-derived compounds such as *C. spinosa* extract demonstrating significant anti-proliferative effects, particularly in colorectal and bladder cancer models (Bacchetti et al. 2022).

In addition to its role in proliferation, PON2 plays a dualistic role in oxidative stress regulation, functioning as an antioxidant shield that maintains cellular redox homeostasis. Overexpression of PON2 protects cancer cells from oxidative damage by ROS, enhancing mitochondrial electron transport chain function and preventing lipid peroxidation (Devarajan et al. 2018; Shakhparonov et al. 2018). HeLa cells overexpressing PON2 are resistant to hydrogen peroxide-induced oxidative stress (Ng et al. 2001), while bladder cancer cells with transient PON2 overexpression show reduced ROS generation upon tert-butyl hydroperoxide treatment (Bacchetti et al. 2017). Conversely, PON2 deficiency markedly increases intracellular ROS levels, sensitizing cells to apoptosis. In lung adenocarcinoma cells, loss of PON2 led to elevated ROS levels and mitochondrial depolarisation, resulting in increased apoptotic susceptibility (Zhao et al. 2016). Melanoma and renal cell carcinoma models similarly show increased oxidative stress upon PON2 knockdown, particularly when combined with chemotherapeutics such as CDDP and 5-FU (Campagna et al. 2020; Schiavoni et al. 2024). Interestingly, certain agents, such as organophosphate pesticides, induce ROS in a PON2-dependent manner. For example, chlorpyrifos and parathion increased ROS levels in neuroblastoma cells, though parathion simultaneously elevated PON2 expression, suggesting a compensatory mechanism (Parween et al. 2022). Similarly, C12-induced ROS and mitochondrial depolarisation were particularly pronounced in PON2-expressing cells (Schwarzer et al. 2015), indicating that PON2 may act as a mediator of specific oxidative stress responses. These findings highlight PON2’s context-dependent antioxidant function: while it predominantly mitigates oxidative stress and promotes tumour survival, certain interactions with bacterial or chemical stimuli can redirect its function towards pro-apoptotic pathways.

PON2’s role in apoptosis is equally complex, involving both protective and sensitizing effects depending on the cellular context. Overexpression of PON2 has been shown to inhibit caspase activation and cell death, as observed in leukaemia and endothelial cell models exposed to imatinib or doxorubicin (Witte et al. 2011). In glioblastoma, PON2 modulation by VPA influenced the expression of apoptosis-related proteins such as Bcl-xL, Bim, and p21, while suppressing migration and invasion (Tseng et al. 2017). Conversely, PON2 silencing or mutation increases apoptosis by promoting caspase-3/7 activation, cytochrome c release, and mitochondrial dysfunction. In renal carcinoma, PON2 knockdown enhanced apoptosis by reducing Ki-67 expression and activating caspase-3 (Schiavoni et al. 2024). In PDAC, PON2 silencing elevated the expression of pro-apoptotic genes such as PUMA and increased annexin V positivity (Nagarajan et al. 2017). Similarly, leukaemia and oral squamous cell carcinoma cells with reduced PON2 expression showed heightened apoptosis, especially after radiation exposure (Krüger et al. 2015, 2016). Notably, PON2’s role in apoptosis also extends to its interaction with bacterial signalling molecules. PON2 has been reported to hydrolyze the bacterial quorum-sensing molecule lactone-3OC12 into cytotoxic products, thereby amplifying apoptosis in certain cell types (Schwarzer et al. 2015). Such findings suggest that PON2’s enzymatic activity can either suppress or promote apoptotic pathways based on external stimuli and intracellular signalling contexts.

Collectively, these findings strongly support the notion that PON2 serves as both a biomarker and a therapeutic target. Elevated PON2 levels are associated with aggressive cancer phenotypes, poor prognosis, and resistance to chemotherapy (Bacchetti et al. 2017, 2021; Pan et al. 2021). Bladder cancer, lung adenocarcinoma, melanoma, leukaemia, and glioblastoma currently show the most robust evidence for PON2 as a therapeutic target, given the strong correlation between PON2 activity, cell survival, and chemoresistance, and the clear effects of its knockdown (Zhao et al. 2016; Bacchetti et al. 2017; Campagna et al. 2020; Pan et al. 2021; Hui et al. 2022; Whitt et al. 2023). Conversely, targeted suppression of PON2 enhances drug sensitivity, reduces proliferation, and promotes apoptosis, making it an attractive candidate for therapeutic intervention. However, translating these findings into clinical strategies presents challenges. PON2 is ubiquitously expressed in normal tissues, where it plays essential roles in maintaining oxidative balance and cellular homeostasis. Therefore, developing selective inhibitors or modulators that target tumour-specific PON2 overexpression without affecting normal tissue function is crucial.

### Future perspectives and challenges

Future research on PON2 should focus on elucidating the upstream regulatory pathways and molecular mechanisms that control its expression and activity across different cancer types. Evidence from current studies suggests that PON2 regulation involves oncogenic signalling pathways such as PI3K/Akt/IKK/NF-κB and tumour suppressors like p53 (Nagarajan et al. 2017; Que et al. 2022), yet a systematic understanding of these networks remains incomplete. Multi-omic strategies integrating genomics, transcriptomics, and metabolomics could provide deeper insights into how PON2 contributes to tumour heterogeneity, progression, and resistance to therapy.

Therapeutically, the development of selective PON2 modulators or inhibitors remains a major challenge due to their ubiquitous expression in normal tissues and their physiological role in maintaining oxidative homeostasis. Structure-guided drug design may help identify compounds that specifically target cancer-associated PON2 overexpression while sparing its protective antioxidant functions in healthy cells. Additionally, plant-derived compounds such as *C. spinosa* extracts (Bacchetti et al. 2022) and pharmacological agents like VPA (Tseng et al. 2017) highlight promising avenues for PON2 modulation, but further optimisation and preclinical validation are required.

From a diagnostic and prognostic standpoint, large-scale clinical studies are needed to validate PON2 as a biomarker of tumour aggressiveness and treatment response. High PON2 expression is consistently linked with poor prognosis and chemoresistance (Bacchetti et al. 2017, 2021; Pan et al. 2021), suggesting its potential use in patient stratification. Combining PON2 profiling with established biomarkers could enhance precision oncology approaches.

Challenges also persist in understanding the dualistic role of PON2 in ROS regulation and apoptosis. While PON2 protects cancer cells from oxidative stress, certain conditions (e.g., exposure to C12 or organophosphates) can redirect its function towards pro-apoptotic pathways (Schwarzer et al. 2015; Parween et al. 2022). This context-dependent behaviour complicates the therapeutic targeting of PON2 and warrants deeper mechanistic studies. Furthermore, the interplay between PON2-mediated redox control, cell proliferation, and apoptosis in therapy-resistant tumours needs exploration to develop synergistic combination treatments that enhance oxidative stress or apoptotic signalling.

Overall, while the therapeutic and diagnostic potential of PON2 is promising, challenges related to its functional complexity, context-dependent roles, and normal tissue expression must be addressed before clinical translation can be achieved.

### Limitations of the study

There remain several limitations to the narrative review investigating PON2 as a cancer biomarker and therapeutic target. The reviewed papers show significant variation in terms of design, sample size, methods, and results. No clinical trials were found during the literature search. Moreover, research findings could have been susceptible to publication bias, incoherent study quality, limited data availability, as well as potential geographical and linguistic biases. The potential for generalisation of the results may be limited by confounding factors such as heterogeneity in patient populations and emphasis on distinct cancer types. The analysis has been rendered challenging considering differences in PON2 measurement methods, possible financing biases, and an absence of long-term outcome data. Acknowledging these constraints appears crucial for offering an equitable analysis and steering future studies.

## Conclusion

PON2 is a multifaceted enzyme with significant roles in cancer biology, spanning proliferation, oxidative stress regulation, and apoptosis. Its overexpression is strongly associated with tumour progression and therapy resistance, while its silencing sensitizes cancer cells to chemotherapeutics and pro-apoptotic stimuli. The dual nature of PON2 underscores its complexity as both a therapeutic target and a biomarker. Continued mechanistic and translational research will be essential to harness the potential of PON2 in cancer diagnostics and treatment strategies.

## Data Availability

No datasets were generated or analysed during the current study.
